# Maximal lymph nodal diameter on N stage of nasopharyngeal carcinoma

**DOI:** 10.1097/MD.0000000000026543

**Published:** 2021-07-02

**Authors:** Shi-Ting Huang, Song Qu, Ling Li, Kai-Hua Chen, Xiao-Dong Zhu, Xin-Bin Pan

**Affiliations:** Department of Radiation Oncology, Guangxi Medical University Cancer Hospital, Nanning, Guangxi, P.R. China.

**Keywords:** maximal nodal diameter, N stage, nasopharyngeal carcinoma

## Abstract

To assess the maximal lymph nodal diameter on the 8th edition American Joint Committee on Cancer staging system of nasopharyngeal carcinoma (NPC).

This study extracted NPC patients between 2004 and 2016 in the Surveillance, Epidemiology, and End Results database. Included patients were divided into 3 groups: ≤3 cm, >3–6 cm, and >6 cm based on the maximal lymph nodal diameter. Cumulative survival curves of 5-year overall survival (OS) and cancer-specific survival (CSS) were calculated using the Kaplan-Meier method between the 3 groups.

The 5-year OS (64.0% vs 59.3%, *P* = .240) and CSS (71.8% vs 67.0%, *P* = .242) of ≤3 cm and >3–6 cm groups were not different. In contrast, the 5-year OS and CSS were different between >6 cm and ≤3 cm groups, and between >6 cm and >3–6 cm groups. The stratified hazard ratio of OS and CSS was 1.75 (95% confidence interval: 1.25–2.45; *P* = .001) and 1.77 (95% confidence interval: 1.20–2.60; *P* = .004) for the >6 cm group in the multivariate regression analysis.

It is reasonable that the maximal lymph nodal diameter with >6 cm is classified as stage N3 of the 8th edition American Joint Committee on Cancer staging system for NPC.

## 1 Introduction

Distant metastasis is the main failure pattern for nasopharyngeal carcinoma (NPC).^[1,2]^ N stage of NPC is the most important predictive factor of distant metastasis. An accurate N stage is crucial to formulate treatment plans and evaluate prognosis. The 8th edition American Joint Committee on Cancer (AJCC) staging system of NPC was proposed.^[3]^ In the 8th edition staging system, the maximal lymph nodal diameter on N stage is divided into 2 groups: >6 cm and ≤6 cm. The maximal lymph nodal diameter with >6 cm is classified as stage N3. Metastatic lymph node with a size >3–6 cm, which was defined as stage N2 in the 2008 Chinese edition staging system is excluded in the 8th AJCC edition.^[3]^ This raises a question of whether survival rates among groups of ≤3 cm, >3–6 cm, and >6 cm are different. Thus, we conducted this retrospective cohort study to assess the maximal lymph nodal diameter on N stage of the 8th edition staging system using data from the Surveillance, Epidemiology, and End Results (SEER) database.

## 2 Patients and methods

### 2.1 Patients cohort

This study extracted NPC cases from 2004 to 2016 in the SEER database. Patients were included when they met the following criteria:

(1) pathologically confirmed NPC;
(2) definite data of maximal lymph nodal diameter could be extracted;
(3) World Health Organization (WHO) type I, II, or III.

Patients with unknown clinical information were excluded. Variables of age, race, sex, WHO classification, tumor grade, radiotherapy, and chemotherapy were extracted. According to the 2008 Chinese edition and the 8th AJCC edition of NPC,^[3,4]^ included patients were divided into 3 groups: ≤3 cm, >3–6 cm, and > 6 cm based on the maximal lymph nodal diameter.

### 2.2 Statistical analysis

Age was transformed to a categorical variable according to a previous study.^[5]^ Categorical variables of age, race, sex, tumor histology, tumor grade, radiotherapy, and chemotherapy were analyzed by using the χ^2^ test or Fisher exact test.

Cumulative survival curves of 5-year overall survival (OS) and cancer-specific survival (CSS) were calculated using the Kaplan-Meier method. Differences between survival curves were compared using the log-rank test. The hazard ratios (HRs) and 95% confidence intervals (CIs) for OS and CSS were estimated with the use of a stratified Cox regression model, with the stratification factors of age, race, sex, WHO classification, tumor grade, radiotherapy, and chemotherapy.

All statistical analyses were performed using SPSS Statistics Version 26.0 software (IBM Co., Armonk, NY) and R software version 4.0.3 (http://www.R-project.org). *P* values were two-tailed. Values of *P* < .05 were considered statistically significant.

Ethical review and approval were waived for this study, due to all data deriving from SEER public databases.

## 3 Results

### 3.1 Patients

**Figure 1:**
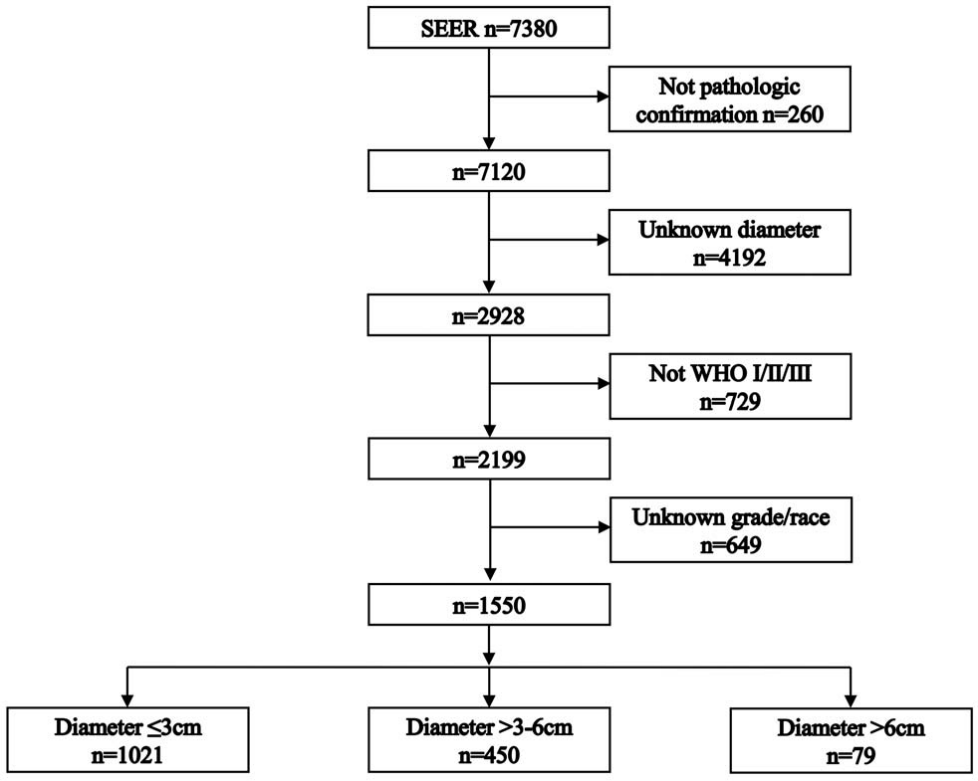
Patient selection flowchart.

Figure 1 shows the process of patient selection. This study included 1550 NPC patients. The patient characteristics were showed in Table 1. Baseline characteristics were well balanced in the variables of age, grade, WHO classification, radiotherapy, and chemotherapy. The median diameter was 2.0 (interquartile range [IQR]: 1.5–2.5), 4.0 (IQR: 3.5–5.0), and 7.0 (IQR: 6.8–8.5) cm of the ≤3 cm, >3–6 cm, and >6 cm groups, respectively.

**Table 1:** Baseline patient characteristics.

	≤ 3 cm (n = 1021)	> 3–6 cm (n = 450)	> 6 cm (n = 79)	*P*
Diameter (cm)				
Median (IQR)	2.0 (1.5–2.5)	4.0 (3.5–5.0)	7.0 (6.8–8.5)	
Age (yr)				
≤19	28 (2.7%)	16 (3.6%)	5 (6.3%)	.277
20–39	136 (13.3%)	67 (14.9%)	12 (15.2%)	
40–59	515 (50.4%)	237 (52.7%)	40 (50.6%)	
60–79	318 (31.1%)	115 (25.6%)	19 (24.1%)	
≥80	24 (2.4%)	15 (3.3%)	3 (3.8%)	
Sex				
Male	703 (68.9%)	338 (75.1%)	65 (82.3%)	.004
Female	318 (31.1%)	112 (24.9%)	14 (17.7%)	
Race				
Asian	410 (40.2%)	200 (44.4%)	43 (54.4%)	.023
Black	114 (11.2%)	51 (11.3%)	12 (15.2%)	
White	497 (48.7%)	199 (44.2%)	24 (30.4%)	
Grade				
I	22 (2.2%)	8 (1.8%)	2 (2.5%)	.191
II	130 (12.7%)	39 (8.7%)	6 (7.6%)	
III	421 (41.2%)	185 (41.1%)	29 (36.7%)	
IV	448 (43.9%)	218 (48.4%)	42 (53.2%)	
Pathology				
WHO I	401 (39.3%)	164 (36.4%)	24 (30.4%)	.451
WHO II	313 (30.7%)	143 (31.8%)	25 (31.6%)	
WHO III	307 (30.1%)	143 (31.8%)	30 (38.0%)	
Radiotherapy				
No	96 (9.4%)	50 (11.1%)	9 (11.4%)	.551
Yes	925 (90.6%)	400 (88.9%)	70 (88.6%)	
Chemotherapy				
No	92 (9.0%)	36 (8.0%)	6 (7.6%)	.771
Yes	929 (91.0%)	414 (92.0%)	73 (92.4%)	

IQR = interquartile range, WHO = World Health Organization.

### 3.2 Overall survival analysis

**Figure 2:**
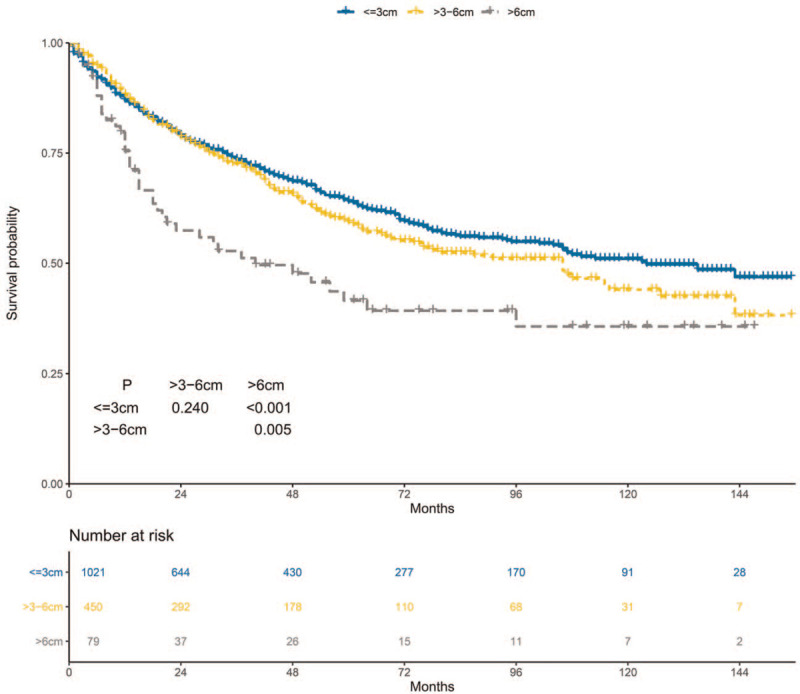
Overall survival of the ≤3 cm, >3–6 cm, and >6 cm groups.

**Figure 3:**
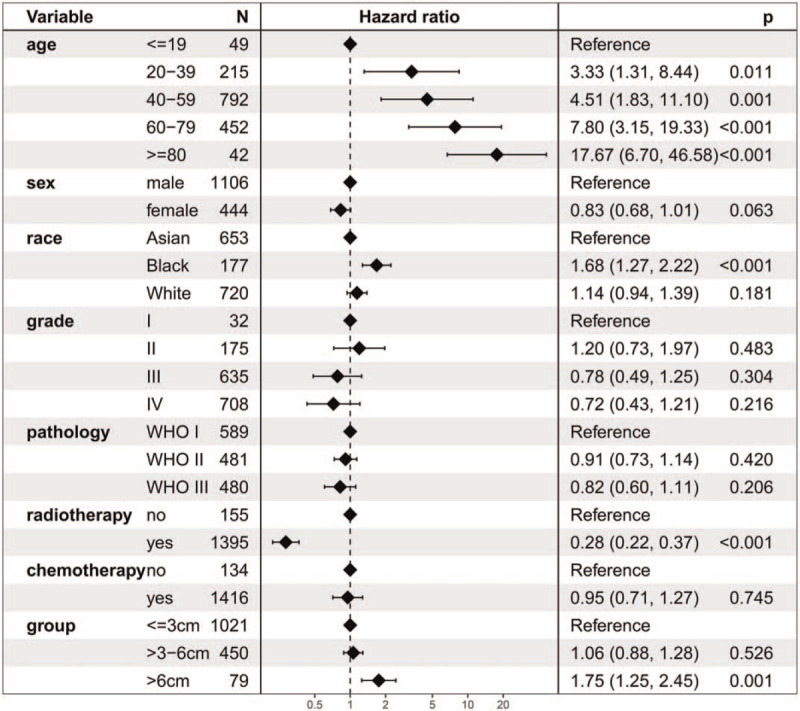
Cox regression analysis for overall survival.

The 5-year OS of the ≤3 cm, >3–6 cm, and >6 cm groups was 64.0%, 59.3%, and 41.5%, respectively (Fig. 2). OS was worse in the >6 cm group than that in the ≤3 cm and >3–6 cm groups. However, difference of OS was not observed between the ≤3 cm and >3–6 cm groups. The stratified HR of the >6 cm group was 1.75 (95% CI: 1.25–2.45; *P* = .001) in the multivariate regression analysis (Fig. 3). In contrast, the stratified HR of the >3–6 cm group was 1.06 (95% CI: 0.88–1.28; *P* = .526).

### 3.3 Cancer-specific survival analysis

**Figure 4:**
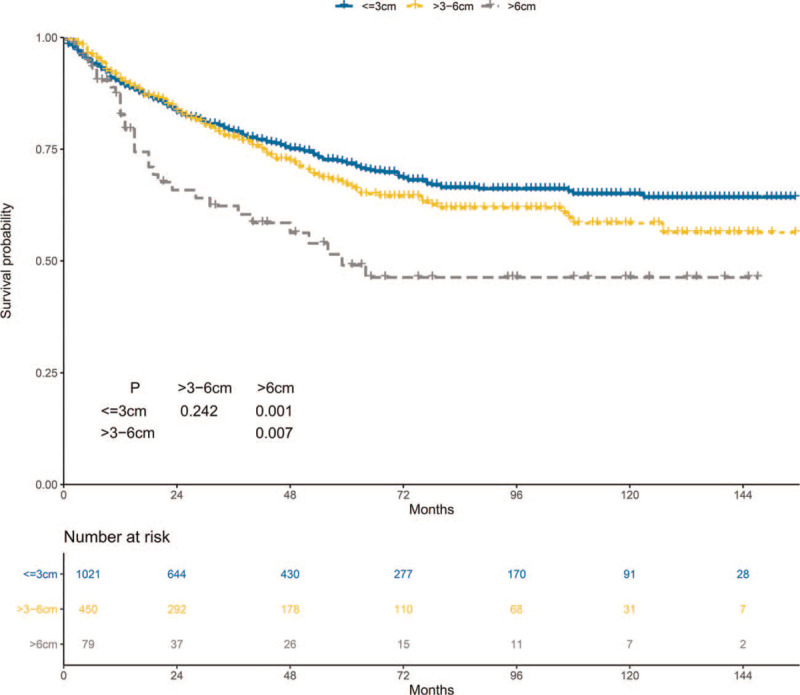
Cancer-specific survival of the ≤3 cm, >3–6 cm, and >6 cm groups.

**Figure 5:**
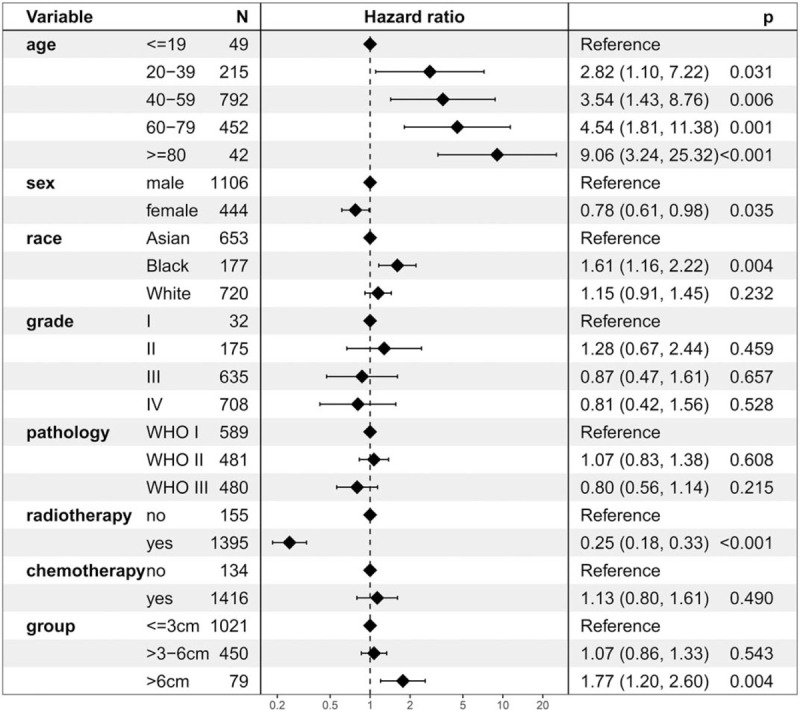
Cox regression analysis for cancer-specific survival.

The 5-year CSS of the ≤3 cm, >3–6 cm, and >6 cm groups was 71.8%, 67.0%, and 49.0%, respectively (Fig. 4). CSS was lower in the >6 cm group than that in the ≤3 cm and >3–6 cm groups. In contrast, CSS was not difference between the ≤3 cm and >3–6 cm groups. The stratified HR of the >6 cm group was 1.77 (95% CI: 1.20–2.60; *P* = .004) in the multivariate regression analysis (Fig. 5). However, the stratified HR of the >3–6 cm group was 1.07 (95% CI: 0.86–1.33; *P* = .543).

## 4 Discussion

Our study assessed the efficacy of the maximal lymph nodal diameter on N stage of the 8th edition staging system. The results revealed that the maximal lymph nodal diameter >6 cm group had worse OS and CSS compared with the ≤6 cm group. Moreover, the 5-year OS and CSS between ≤3 cm and >3–6 cm groups were not different. These results suggested that lymph nodal size with >6 cm as N3 criteria of the 8th edition AJCC staging system for NPC was reasonable.

However, the maximal lymph nodal diameter >6 cm as N3 criteria is still not well investigated. Pan et al^[3]^ reported that distant metastasis-free survival and OS were significantly different between stage N3 and N2 of the 8th edition AJCC staging system. However, several studies suggested that lymph nodal size with >6 cm was not an independent prognostic factor.^[6–10]^ The potential interpretations for the differences among previous studies may be the following:

(1) The proportion of lymph nodal size with >6 cm is small, which was less than 4.5%.^[3,6–8]^ The small sample size of lymph nodal size with >6 cm group might significantly reduce the statistical power of the analysis.
(2) The N classification of AJCC staging system defines lymph nodal size by the largest dimension, irrespective of the measurement plane. This measurement was considered the best surrogate of gross lymph nodal volume. However, several studies defined the maximal lymph nodal diameter based on cross-sectional imaging.^[6,7,11]^ The lymph nodal diameter in cross-sectional imaging might not be the maximal diameter.

Moreover, the efficacy of metastatic lymph nodes size with >6 cm as N3 criteria should be further identified. First, maximal lymph nodal diameter >6 cm as N3 criteria was established in the 6th edition AJCC staging system. The maximal lymph nodal diameter of the 6th and 7th edition AJCC staging system is mainly based on palpation. The measurement differs among clinicians.^[12]^ Second, the maximal lymph nodal diameter is mainly defined based on magnetic resonance imaging. The optimal cut-off value of metastatic lymph nodes size as N3 criteria needs to assess using a more reliable algorithm. Third, the tumor volume^[13,14]^ and metabolic tumor volume^[15–18]^ may be better factors than the metastatic lymph nodes size in representing the tumor burden. These factors might replace the maximal lymph nodal diameter in further staging systems.

According to our study, it was reasonable that metastatic lymph nodes with a size >3–6 cm was not classified as stage N2 in the 8th edition AJCC staging system. The Cox regression analysis showed that metastatic lymph nodes with a size >3–6 cm was not an independent prognostic factor for CSS and OS. Similarly, several studies revealed that metastatic lymph nodes with a size >3–6 cm failed to achieve an independent prognostic factor in survivals.^[3,6–8]^

This study had a limitation. Data of distant metastasis could not be extracted due to the limitations of SEER database. It was reported that the major failure pattern of NPC was distant metastasis.^[1,2]^ N stage was the most important predictive factor of distant failure. Thus, this study could not assess the distant-metastasis free survival among groups of ≤3 cm, >3–6 cm, and >6 cm. Whether the worse OS and CSS with metastatic lymph nodes size >6 cm were due to distant failure or not was still unknown. In further, more studies are needed to assess the association between metastatic lymph nodes size and distant failure.

In conclusion, it is reasonable that lymph nodal size with >6 cm is classified as stage N3 of the 8th edition AJCC staging system for NPC.

## Author contributions

**Conceptualization:** Shi-Ting Huang, Xin-Bin Pan.

**Data curation:** Shi-Ting Huang.

**Formal analysis:** Shi-Ting Huang, Ling Li.

**Methodology:** Song Qu, Xiao-Dong Zhu.

**Software:** Song Qu.

**Validation:** Ling Li, Kai-Hua Chen, Xiao-Dong Zhu.

**Writing – original draft:** Xin-Bin Pan.

**Writing – review & editing:** Xin-Bin Pan.
